# Technology acceptance and depressive symptoms among community-dwelling older people

**DOI:** 10.1093/geroni/igab046.3143

**Published:** 2021-12-17

**Authors:** Suk-Young Kang, Jeungkun Kim, Jeffrey Winthal, Rosemarie Lenz

**Affiliations:** 1 Binghamton University, State University of New York, Binghamton, New York, United States; 2 Kangnam University, Yong-In, Kyonggi-do, Republic of Korea; 3 Binghamton University, Binghamton, New York, United States

## Abstract

Depression is a major public health issue among older adults, with an estimated prevalence between 5% and 10%. The aim of this study is to explore the possible benefits technology acceptance has in reducing depression among older people. Mail-survey data were collected from community-dwelling adults over the age of 65. This method was chosen over face-to-face surveys due to Covid-19. There were 192 total participants. The GDS-5 was used to measure the level of depressive symptoms. Among the participants, 25 participants (13%) scored higher than 2, indicating the presence of depression. Using a hierarchical regression analysis revealed that the equation explained 42.4 % of the variance (adjusted R square =.382) in levels of depression (F (3,176) = 9.973, p <.000). Variance inflation factor (VIF) values were smaller than 10, indicating that multicollinearity among the correlates was not an issue. The correlates of the level of depression were: level of education, overall physical health, level of loneliness, perceived ease of technology use, attitude toward technology use, and intent to purchase new technology for older people. Results indicate that a positive attitude toward technology use might be inversely associated with depression levels. This shows how quality of life related to mental health may be improved by a change in attitude in favor of technology use. Participants were interested in learning to use new technology, and would like more opportunities to do so. Policy changes that increase lifelong learning options would help to make this happen.

